# Widespread distribution of prophage-encoded virulence factors in marine *Vibrio* communities

**DOI:** 10.1038/s41598-018-28326-9

**Published:** 2018-07-02

**Authors:** Daniel Castillo, Kathryn Kauffman, Fatima Hussain, Panos Kalatzis, Nanna Rørbo, Martin F. Polz, Mathias Middelboe

**Affiliations:** 1 Marine Biological Section, University of Copenhagen, Strandpromenaden 5, DK-3000 Helsingør, Denmark; 2 Department of Civil and Environmental Engineering, Massachusetts Institute of Technology, 77 Massachusetts Avenue, Cambridge, MA 02139 USA

## Abstract

Prophages are known to encode important virulence factors in the human pathogen *Vibrio cholerae*. However, little is known about the occurrence and composition of prophage-encoded traits in environmental vibrios. A database of 5,674 prophage-like elements constructed from 1,874 *Vibrio* genome sequences, covering sixty-four species, revealed that prophage-like elements encoding possible properties such as virulence and antibiotic resistance are widely distributed among environmental vibrios, including strains classified as non-pathogenic. Moreover, we found that 45% of *Vibrio* species harbored a complete prophage-like element belonging to the *Inoviridae* family, which encode the zonula occludens toxin (Zot) previously described in the *V*. *cholerae*. Interestingly, these *zot*-encoding prophages were found in a variety of *Vibrio* strains covering both clinical and marine isolates, including strains from deep sea hydrothermal vents and deep subseafloor sediments. In addition, the observation that a spacer from the CRISPR locus in the marine fish pathogen *V*. *anguillarum* strain PF7 had 95% sequence identity with a *zot* gene from the *Inoviridae* prophage found in *V*. *anguillarum* strain PF4, suggests acquired resistance to inoviruses in this species. Altogether, our results contribute to the understanding of the role of prophages as drivers of evolution and virulence in the marine *Vibrio* bacteria.

## Introduction

The *Vibrio* genus (vibrios) is a genetically and metabolically diverse group of heterotrophic bacteria that are ubiquitously distributed in the oceans and often accounts for a large fraction (0.5–5%) of the total bacterial community, occasionally developing massive blooms^1,2^. The group includes several human pathogens (e.g., *V*. *cholerae* and *V*. *parahaemolyticus*)^3^, and pathogens infecting corals (*V*. *coralliilyticus*) and fish (e.g., *V*. *anguillarum*, *V*. *harveyi*)^4,5^. Virulence-related factors in these pathogens are often associated with mobile genetic elements (e.g., prophages and genomic islands)^6,7^, suggesting that horizontal acquisition of these elements is required for the development of virulence in vibrios. A well-known example of prophage-associated virulence is in *V*. *cholerae*^8^ where the key toxins (CTX and Zonula occludens toxin (*zot*)) are prophage encoded. The *zot* gene was first detected in *V*. *cholerae* and is encoded by the CTXφ prophage^9^. Besides its role as a cytotoxin, Zot appears to be structurally and functionally related to CTX phage assembly, suggesting that this protein may have a dual function^10^.

Prophage-associated toxins have also been identified in the marine vibrios such as: *V*. *coralliilyticus*^11^ and *V*. *anguillarum*^7^ and *V*. *parahaemolyticus*^12^, indicating that prophage-encoded virulence genes are disseminated among environmental *Vibrio* populations. Recently, a *zot*-encoding prophage was detected in three fish pathogenic*V*. *anguillarum* strains^7^. The Zot-like protein contained an N-terminus involved in phage assembly, and C-terminal, which is cleaved and secreted into intestinal lumen where it increases intestinal epithelial permeability by affecting the tight junctions^9^. Similarly, prophage genomes encoding toxin genes with homology to those found in pathogenic *V*. *cholerae* were found in *V*. *coralliilyticus* genomes^11^. Also, experimental evidence of efficient prophage-mediated transfer of virulence between strains of the fish pathogen *V*. *harveyi*^12^ emphasizes that prophage-encoded virulence is a dynamic property of environmental vibrios. Together, these studies strongly suggest that prophages in *Vibrio* bacteria harbor multiple and diverse genetic elements with large effects on pathogenicity and host fitness and a large potential for dissemination among the *Vibrio* group. Thus, the emergence of environmental *Vibrio* bacteria with significant virulence traits constitutes a direct concern for human public health, food, and the aquaculture industry. While the ability of prophages to impact the pathogenicity of *V*. *cholerae* is well studied^8,13^, little is known about the distribution and role of prophage-encoded fitness factors in a broader range of environmental *Vibrio* bacteria. We are therefore only beginning to comprehend the extent of prophage influence on microbial performance, gene exchange and evolution in the *Vibrio* group, and the potential of prophage genes as a reservoir of transmissible virulence factors among vibrios in marine environments.

The aim of this study was to explore the potential role of prophages for the dissemination of virulence and other niche adaptation factors in environmental marine vibrios. By mapping and characterization of prophage-like elements and their distribution in the ~2000 available whole genome sequenced *Vibrio* strains, we demonstrate that prophage-encoded functional properties such as virulence and antibiotic resistance are widely distributed among environmental vibrios. Hence, *Vibrio* prophages and temperate phages are potentially key driving forces in niche adaptation, dissemination of virulence and emergence of disease in environmental marine *Vibrio* communities.

## Results

### General features of *Vibrio* prophage-like elements database

**Figure 1:**
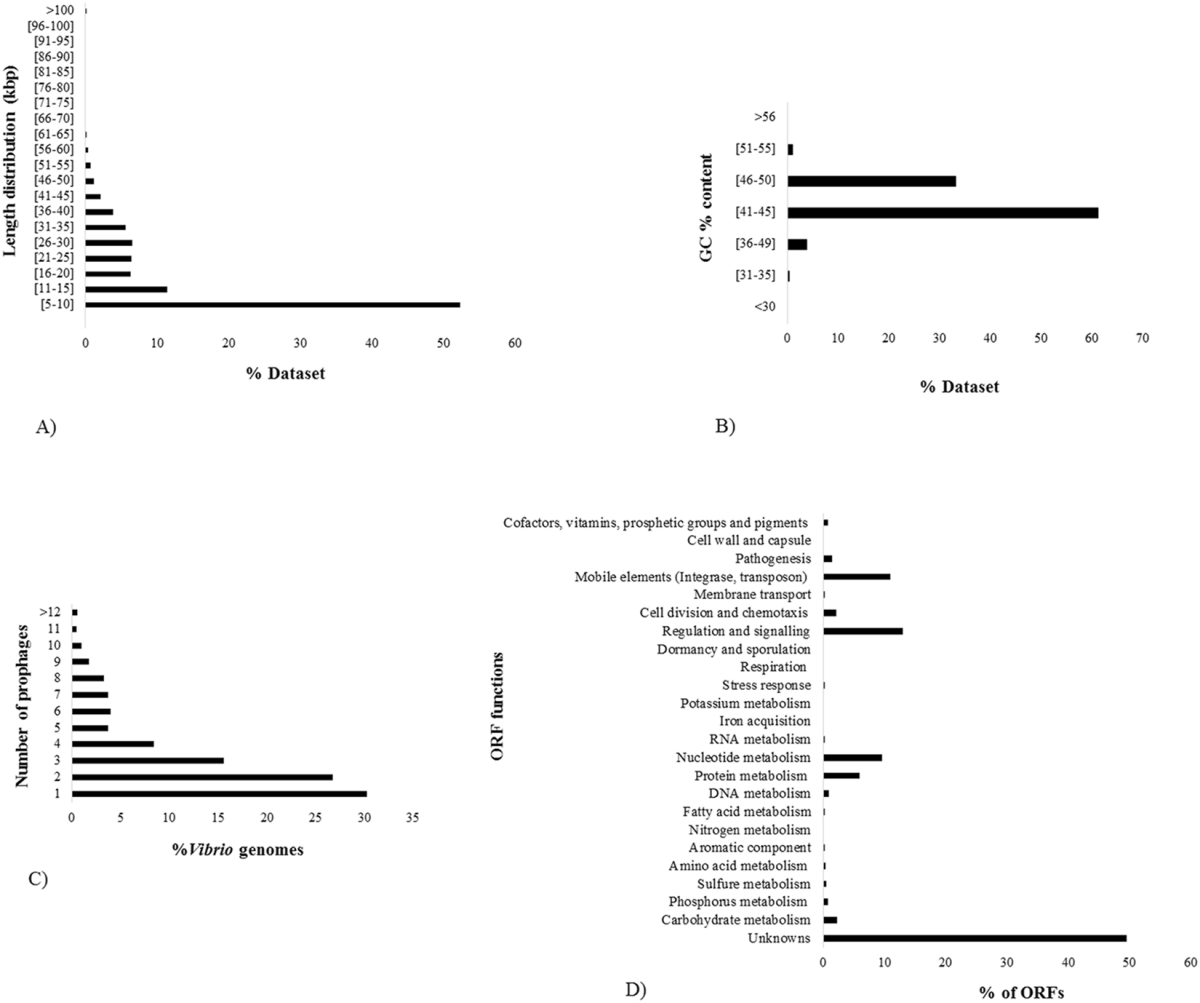
Prophage-like element features among *Vibrio* genomes sequences used in this study. (**A**) length distribution of *Vibrio* prophage-like elements. (**B**) GC% content in *Vibrio* prophage-like elements. (**C**) Distribution of number of prophages per *Vibrio* genome. (**D**) Percentage of annotated functions of ORFs in prophage-like elements (analyzed by SEED Subsystems).

Among the 1,874 sequenced *Vibrio* genomes available in NCBI database (Table S1), we identified the occurrence, composition and distribution of 5,674 prophage-like elements (>10^8^ bp; >90,000 ORFs) representing the largest collection of prophage-like elements in *Vibrio* bacteria so far. Since the available genome sequences of *Vibrio* temperate phages in NCBI database were all >5 kb, we restricted our search to prophage-like elements larger than 5 kb. In general, prophage sequence length and GC content ranged from 5 to 126 kb and 31.7 to 54.9%, respectively. Most of these prophage-like elements had a GC content between 41 and 45% (60%) and a size range of 5–10 kb (54%), corresponding to the smallest sized inovirus prophage sequence available in the NCBI database (Fig. 1A,B; See Supplementary Table S1). Among the 1,874 *Vibrio* genomes, 69.5% were poly-lysogenic, containing >1 prophage sequence (Fig. 1C). Functional analysis of annotated prophage-like elements showed that 49.6% of ORFs were hypothetical proteins without function assigned, while the remaining ORFs represented mostly mobile elements (eg., integrases, tranposases), nucleotide and protein metabolism, regulation and signaling (Fig. 1D). Finally, the completeness of the prophage-like elements was estimated by PHASTER, resulting in 36% intact, 56% incomplete and 8% questionable.

### *Vibrio* prophage-like elements encoded potential host fitness factors

**Figure 2:**
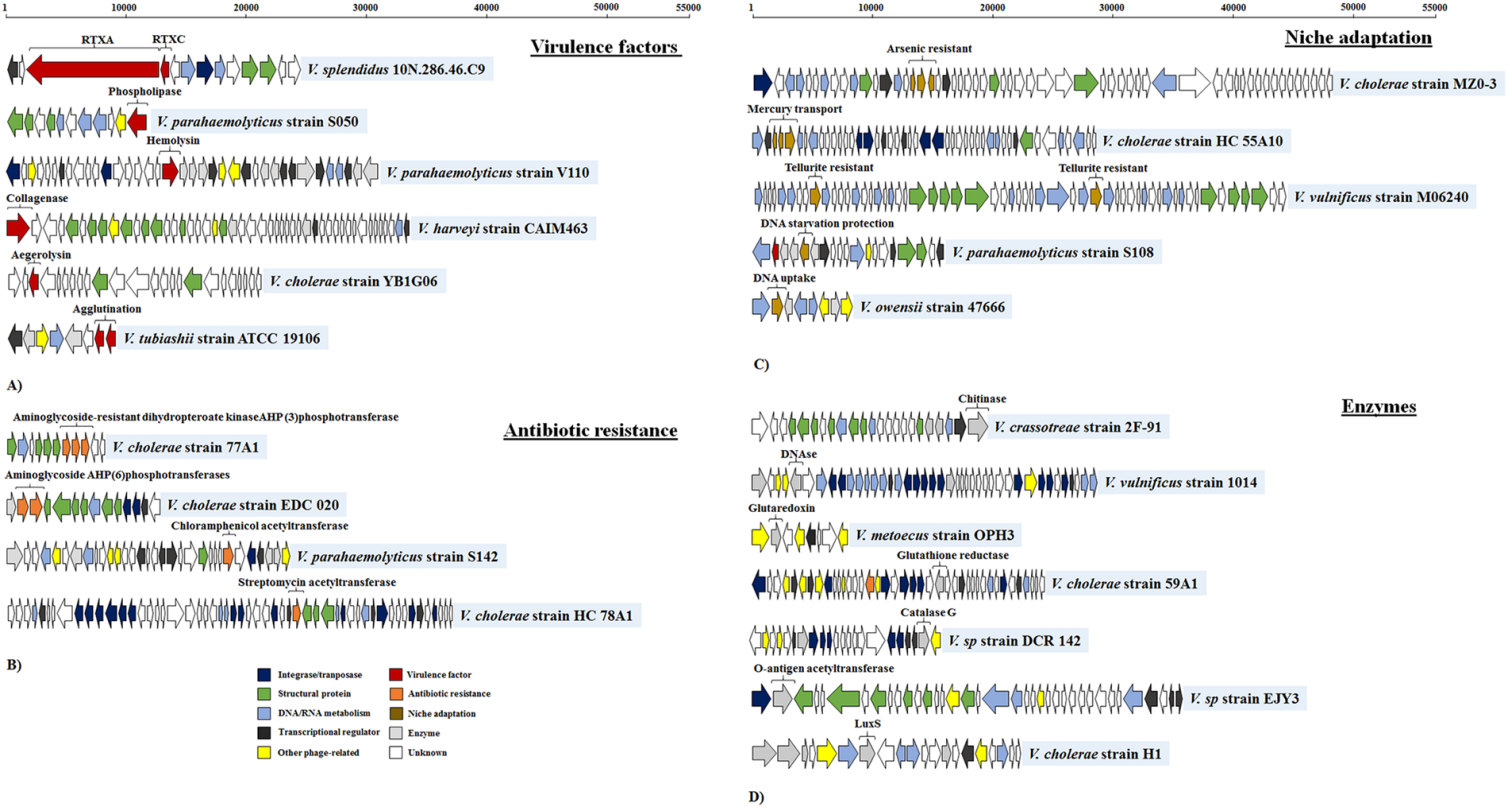
Genomic organization of prophage-like elements detected in *Vibrio* genomes carrying genes potentially beneficial for the host. (**A**) Prophage-like elements carrying virulence factor genes. (**B**) Prophage-like elements carrying antibiotic resistance genes. (**C**) Prophage-like elements carrying niche adaptation genes. (**D**) Prophage-like elements carrying enzyme genes. Arrows represent the predicted ORFs and point in the direction of transcription. The colors were assigned according to the possible role of each ORF as is shown in the figure. The representative phages displayed in this figure have been classified as complete or questionable prophages (See Supplementary Table S2).

In order to assess the potential influence of prophages on host functional properties, we mapped the distribution of key genes could be related to virulence factor production, antibiotic resistance, niche adaptation factors, metabolism and enzymes, according to the annotation pipeline showed by MG-RAST (See Supplementary Table S2). We found that 19.5% (n = 1,109) of the prophage-like elements encoded a potentially important gene for the host. For example, virulence factors such as RTX toxins (n = 100), collagenases (n = 5), lipases (n = 10), agglutination (n = 30), hemolysin (n = 4) and aerolysin (n = 1) were found in prophage like elements belonging to *V*. *parahaemolyticus*, *V*. *cholerae*, *V*. *harveyi*, *V*. *splendidus*, *V*. *tasmaniensis*, *V*. *tubiashii* and *V*. *hepatarius* (Fig. 2A; See Supplementary Table S2). Genes related to antibiotic resistance (total n = 116) (kanamycin, chloramphenicol, streptomycin among others) were found mostly in prophage like-elements in *V*. *cholerae* and *V*. *parahaemolyticus*. We identified genes encoding APH(3″)/APH(6″) family aminoglycoside O-phosphotransferases (n = 3), type B chloramphenicol O-acetyltransferase (n = 23) and streptomycin 3′-adenylyltransferase (n = 5) (Fig. 2B; See Supplementary Table S2). Prophages containing niche adaptation factors with high similarity to heavy metal resistance (n = 32) and natural DNA uptake transformation (n = 3) genes were also found (Table S2). For example, *V*. *cholerae* strains had prophage-like elements encoding genes involved in resistance to arsenic and mercury, while *V*. *vulnificus* harbored a prophage which encoded genes related to tellurite resistance (Fig. 2C; See Supplementary Table S2). *V*. *anguillarum* and *V*. *parahaemolyticus* had prophages encoding the *dps* gene, which is related to DNA protection under starvation conditions (Fig. 2C; See Supplementary Table S2). Finally, *V*. *crassostreae*, *V*. *owensii* and *V*. *caribbeanicus* had prophages that encoded *dprA* gene, which has been linked to natural DNA uptake in aquatic environments (Fig. 2C; See Supplementary Table S2).

Besides the mentioned fitness factors, a sub-classification of enzymes was encoded by a number of *Vibrio* prophages. Chitinases (n = 6) were found mostly in prophage-like elements in *V*. *crassostreae* and *V*. *parahaemolyticus* (Fig. 2D). The presence of *tatD* gene (n = 11), which possesses DNAse activity, was found in pathogenic species including *V*. *vulnificus*, *V parahaemolyticus* and *V*. *cholerae* (Fig. 2D; Table S2). Also, we found redox enzymes such as glutaredoxin (n = 54) and glutathione reductase (n = 19), which were widely distributed in *V*. *metoecus*, *V*. *cholerae*, *V*. *harveyi*, *V*. *navarrensis* and *V*. *alginolyticus* (Fig. 2D; See Supplementary Table S2). Contrarily, catalase KatG (n = 1) was found only in *V*. sp (Fig. 2D). Finally, gene *oafA* (n = 11), which encodes a peptidoglycan/LPS-O-acetylase, was found in prophages of *V*. *alginolyticus*, *V*. *harveyi* and *V*. sp (Fig. 2D). Interestingly, we found a prophage-like element in *V*. *cholerae* encoding *luxS* gene (n = 1), which is involved in the synthesis of the quorum sensing molecule AI-2 (Fig. 2D; See Supplementary Table S2).

### Distribution of Zot-like toxin in *Vibrio* genomes

The zonula occludens toxin gene (*zot*) located on CTX prophage is associated with the pathogenicity of *V*. *cholerae*^9^. In order to examine whether *zot-* encoding prophages are more widely distributed in *Vibrio* species beyond *V*. *cholerae*, we examined the frequency of *zot*-encoding prophages in the entire database and determined the phylogenetic relationship of the Zot toxin amino acid sequence. In addition to 314 Zot toxins found in the CTX prophages of *V*. *cholerae*, we found 501 *zot*-encoding prophages in twenty-eight out of sixty-four *Vibrio* species (14.5% of prophage-like elements database) (See Supplementary Table S1). For these prophages, the %GC ranged from 39.5 to 47.5 and the length from 5–12 kb.

The *zot*-encoding prophages were found in the majority of the clinical isolates of *V*. *cholerae* (56.3%) and *V*. *parahaemolyticus* (77.9%) (See Supplementary Table S1). However, we also found this specific prophage in *Vibrio* species that were not associated with clinical samples or disease outbreaks (Table S1). Overall, 15.2% of environmental marine *Vibrio* isolates contained a *zot*-encoding prophage, including isolates from coastal marine waters (e.g. *V*. *campbellii*), deep hydrothermal vents (e.g. *V*. *antiquarius* and *V*. *diabolicus*) and deep subseafloor sediments (e.g. *V*. *diazotrophicus*) (See Supplementary Table S1).

**Figure 3:**
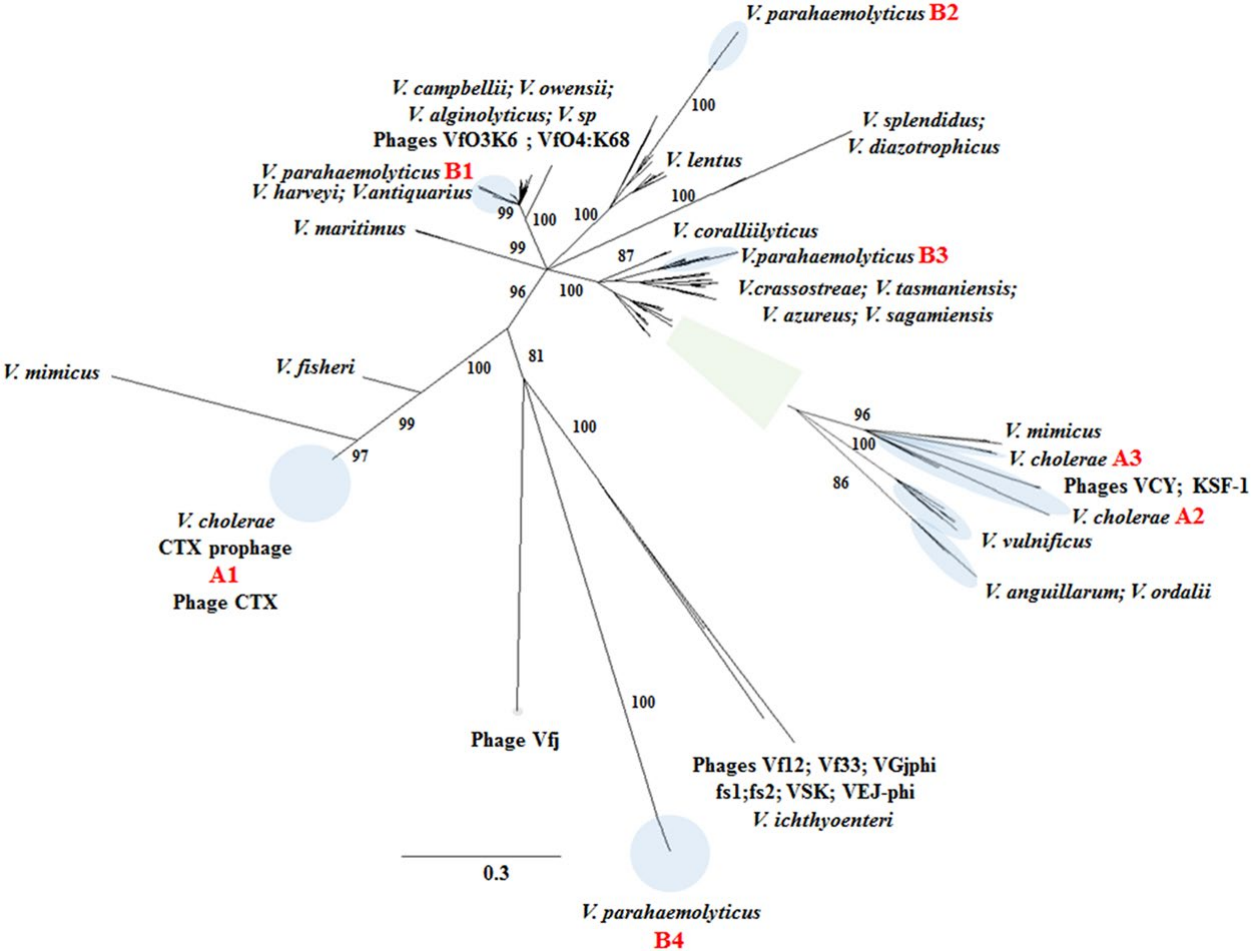
Phylogeny of Zot-like proteins obtained from *zot*-encoding prophages. Unrooted phylogenetic tree constructed from Zot-like toxin amino acid sequences, using the maximum likelihood algorithm with 1000 bootstraps. Bootstrap values <80% were removed from the tree. Circles with a blue color highlight the different phylogenetic groups described in the text (A1–A3 and B1–B4). In addition, green square is a zoom in on the specific cluster in the phylogenetic tree containing the groups A2 and A3. The horizontal bar at the base of the figure represents 0.3 substitutions per amino acid site.

**Figure 4:**
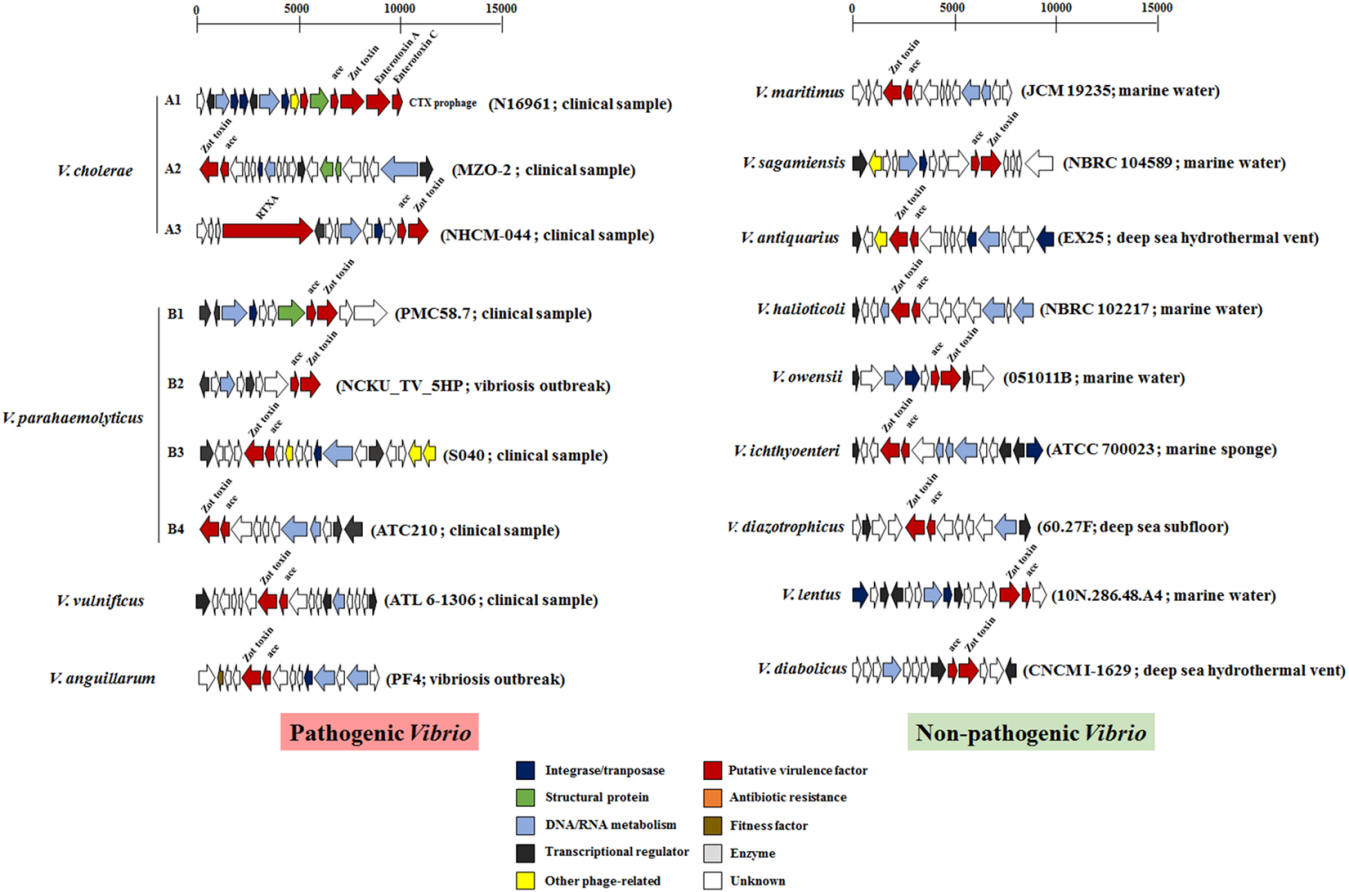
Genomic organization of *zot*-encoding prophages in *Vibrio* species. Diagrammatic representation of *zot*-encoding prophage-like elements integrated into the chromosomes of different pathogenic and non-pathogenic (environmental) *Vibrio* species (See Supplementary Table S1). The colors were assigned according to the possible role of each ORF as is shown in the Figure. Parentheses represent strain name and source of isolation for each example.

Phylogenetic analysis of Zot-like toxin proteins based on maximum likelihood algorithm showed that sequences from *V*. *parahaemolyticu*s and *V*. *cholerae* clustered in subgroups (Fig. 3). *V*. *cholerae* Zot-like toxin grouped in 3 different clusters named A1, A2 and A3. The group A1 consisted of the Zot-like sequences associated with well-studied CTX prophage, whereas the diversity of A2 group differed from the CTX prophage, and A3 contained of group of prophages which had both Zot and RTXA toxins (Figs 3 and 4; Table S2). Similarly, *V*. *parahaemolyticus* displayed four different clusters of Zot-like proteins (B1–B4), where the B4 group included the zot toxin encoded by prophage O3:K6 identified in the pandemic *V*. *parahaemolyticus* clone (Figs 3 and 4). The presence of Zot-like proteins in environmental *Vibrio* species, which have previously been isolated as non-pathogenic strains from several marine environments, included *zot*-encoding prophages in *V*. *maritimus*, *V*. *sagamiensis*, *V*. *owensii*, *V*. *diazotrophicus* and *V*. *halioticoli* (Fig. 4; See Supplementary Table S1). For some *Vibrio* species, the different Zot-like proteins were located in the same phylogenetic group. For example, Zot-like toxins from *V*. *campbellii*, *V*. *owensii*, and *V*. *alginolyticus* grouped together as did the zot toxins in *V*. *anguillarum* and *V*. *ordallii* (Fig. 3). In other *Vibrio* species, the Zot proteins formed monophylogenetic groups, such as in *V*. *vulnificus* and *V*. *coralliilyticus* (Fig. 3). Another feature of this analysis is the divergent clustering of the Zot-like proteins from the documented *Vibrio* filamentous phages (See Supplementary Table S1). Based on the phylogenetic tree, *V*. *parahaemolyticus* phage Vf33^14^ appears to be more closely related to *V*. *cholerae* phages VGJ^15^, fs1^16^, fs2^17^, VSK^18^, VEJ^19^ and VFJ^20^ than with *V*. *parahaemolyticus* filamentous phages VfO3:K6 and VfO4:K68^21^, which made up a distinct branch in the phylogenetic tree (Fig. 3). Interestingly, all these *zot*-encoding prophages contained the Accessory Cholera Enterotoxin gene (*ace*), which has been described also in CTX prophage^9^ (Fig. 4).

**Figure 5:**
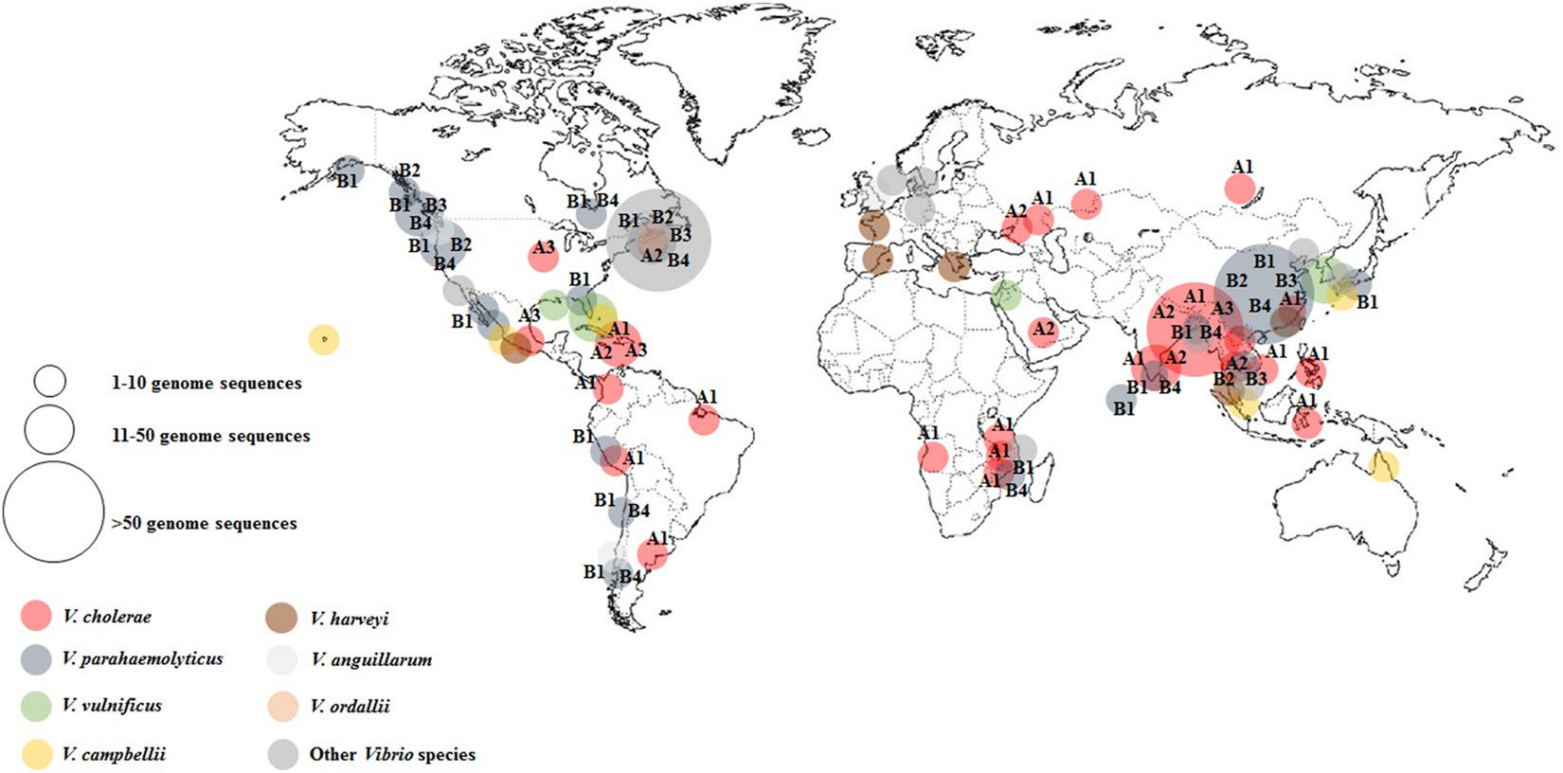
Geographical distribution of *zot*-encoding prophages in *Vibrio* species. Global distribution of *zot*-encoding prophages in *Vibrio* species. Color blocks represent the *Vibrio* species most abundant in our database. The size of the circles is proportional to the number of *Vibrio* genome sequences carrying a *zot*-encoding prophage in a specific geographic location. Main phylogenetic groups identified for *V*. *cholerae* (A1–A3) and *V*. *parahaemolyticu*s (B1–B4) were included in the figure to facilitate comparison.

To include the geographical variability in *zot*-encoding prophages, we determined the spatial distribution of *Vibrio* sequences carrying the prophages. The analysis showed a global scale distribution of Zot-like proteins with no well-defined geographical patterns (Fig. 5). However, some phylogenetic groups were associated with specific geographic sites. For example, *V*. *parahaemolyticus* isolates carrying a Zot-like toxin isolated in India and Bangladesh were linked only to phylogenetic groups B2 and B4. Similarly, Zot-sequences belonging to the group B1 were found mostly along the Pacific coast (Fig. 5).

### CRISPR arrays in *V*. *anguillarum*

**Figure 6:**
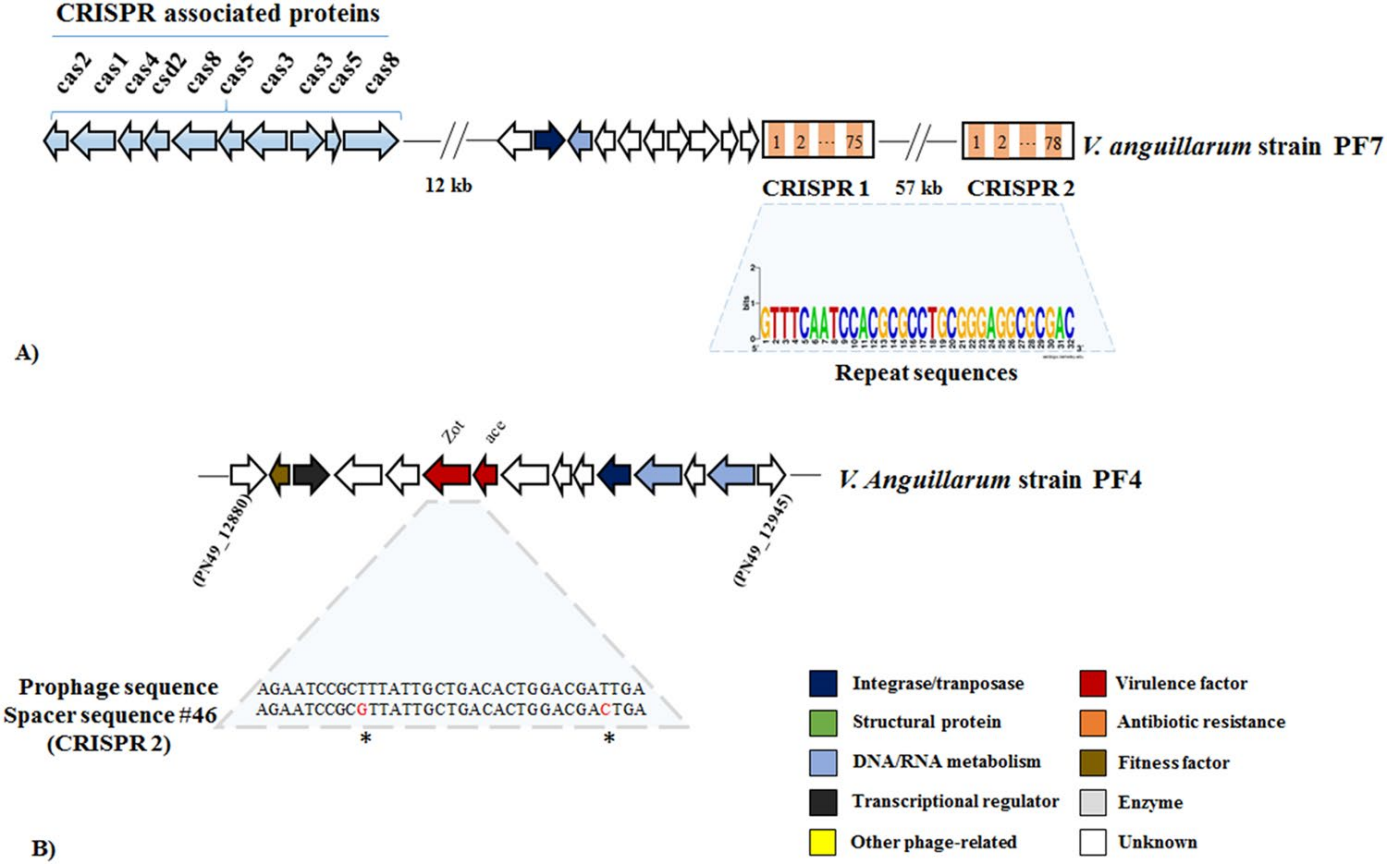
Genomic architecture of CRISPR-Cas systems in *V*. *anguillarum* strain PF7 and potential protection against inoviruses. (**A**) Location and genomic organization of two CRISPR arrays in *V*. *anguillarum* strain PF7. The weblogo represents the conservation of that nucleotide at each position in the repeat. (**B**) Genomic organization of putative *Inoviridae* prophage-like element in *V*. *anguillarum* strain PF4. Comparison of spacer from CRISPR 2 and zot sequence is shown. Asterisks represent point mutations between the prophage sequence and the CRISPR spacer. Arrows display the predicted ORFs and point in the direction of transcription. The colors were assigned according to the possible role of each ORF as is shown in the figure.

Clustered regularly interspaced short palindromic repeats (CRISPR) arrays were previously found in 11.9% of the *Vibrio* genome sequences. In addition, 0.03% of the genomes had more than one CRISPR array^22^, and the longest array found in *V*. *anguillarum* strain PF7 (Accession numbers: CP011464 and CP011465) harbored ten *cas* genes (type I CRISPR-cas system) with identical repeats of 32 bp and two arrays with seventy-eight and seventy-five spacers, respectively (Fig. 6A; 14). Comparing nucleotide similarity of *V*. *anguillarum* spacers from these two CRISPRs with sequences of all *zot*-encoding prophage-like elements in our database revealed that 1 of 153 spacers (CRISPR array 2, spacer #46) matched (95% similarity) a *zot* toxin gene in a potential *Inoviridae* prophage sequence from *V*. *anguillarum* strain PF4 (Accession numbers: CP010080 and CP010081) (Fig. 6B).

## Discussion

Despite the well-established role of prophages as sources of virulence properties in vibrios, little is known about the abundance, genetic composition and diversity, and induction abilities of prophage-like elements in *Vibrio* species beyond human pathogens such as *V*. *cholerae*^8,9^. Exploring the *Vibrio* prophage database for the presence of specific genes that potentially confer virulence or niche adaptations to the host demonstrated that these properties were widely distributed in the global collection of sequenced genomes (Fig. 2; See Supplementary Table S2). Specifically, our results showed that virulence, niche adaptation and antibiotic resistance genes encoded by prophage-like elements are not restricted to human pathogens (e.g. *V*. *cholerae* and *V*. *parahaemolyticus*) but are efficiently exchanged among the *Vibrio* genus, and dispersed among species considered to be non-pathogenic such as *V*. *azureus*, *V*. *hepatarius* and *V*. *diazotrophicus* (Figs 2–4). One of the *V*. *diazotrophicus* isolate harboring a *zot*-encoding prophage originated from sediments 79.5 meter below seafloor, corresponding to an estimated sediment age between 16,000–80,000 years^23,24^, indicating the that these prophages have been interacting with their *Vibrio* hosts across long term evolutionary time scales. Moreover, 49.6% of the phage-encoded ORFs in the database were predicted to encode hypothetical proteins with unknown functions (Fig. 1D), which potentially also could carry out functions supporting host performance and adaptations^25^. Thus, *Vibrio* prophages and temperate phages may constitute a major reservoir of virulence and niche adaptation traits in marine systems. Additionally, their widespread distribution among *Vibrio* species suggests that the induction and integration of these phages drive the dissemination of these genes, contributing to the genetic diversification and functional adaptations of *Vibrio* communities^26^.

In addition to *V*. *cholerae*, several other *Vibrio* species were recently reported to contain *zot*-encoding prophages^7,24,27^. Here we found a cosmopolitan distribution of *zot*- encoding prophages in twenty-eight different *Vibrio* species such as *V*. *vulnificus*, *V*. *maritimus*, *V*. *azureus*, *V*. *crassostreae*, *V*. *diazotrophicus* and *V*. *halioticoli* (Fig. 4; See Supplementary Table S1). This is in line with the large-scale distribution of *zot*-encoding prophages across the world’s oceans, suggesting the interaction and co-existence of inovirus phages and *Vibrio* bacteria on a global scale (Fig. 5)^28^. Thus, our data suggest that *zot*-encoding prophages are widespread in *Vibrio* species and define a group of prophage-like elements that could undergo extensive horizontal gene transfer. This suggestion is supported by the phylogenetic analysis, where many of the Zot-like proteins from the different *Vibrio* species (e.g., *V*. *owensii* and *V*. *alginolyticus*; *V*. *splendidus* and *V*. *diazotrophicus*) showed high similarity, despite very different genomic organization of the prophages they originated from (Fig. 4). The fact that many variants of *zot*-encoding prophages are prevalent in specific species suggests that they could play a role in horizontal transfer of the *zot* gene, as has been reported for CTX and other filamentous phages^19,21^. Moreover, our results displayed a wide geographical distribution of *zot*-encoding prophages, suggesting also that these specific elements may have an important role in the dispersion of genes by exploring new *Vibrio* hosts (Fig. 5)^7,24,27^. Interestingly, we demonstrate that specific lineages of the Zot toxin are not exclusively associated with specific pathogenic species, again suggesting that lysogenization with this group of phages occurs across the *Vibrio* genus.

Lysogenic conversion of non-virulent strains into virulent ones by integration of a prophage has so far mainly been associated with the emergence of new virulent and epidemic clones of human pathogens such as *V*. *cholerae* and *E*. *coli* O157:H7 following lysogenization with phage CTXφ^8^ and the shiga toxin encoding phages Sp5 and Sp15^29^, respectively. Thus, the suggested efficient exchange and dispersal of virulence factors among environmental *Vibrio* species represents a novel perception of prophages as drivers of the virulence evolution and diversification of the global *Vibrio* communities, and supports recent evidence that virulence in the coral pathogen *V*. *coralliilyticus* is acquired by lysogenization with *zot*-encoding prophages^10^. The current observation that several *Vibrio* species designated as harmless environmental bacteria contained specific virulence traits acquired from induced phages from pathogenic donors, suggests that these strains act as potential biological reservoirs of these genes in the environment (Fig. 4). This idea is supported by observations in *V*. *mimicus*, which harbor CTX phage and may play a role in the emergence of new pathogenic *V*. *cholerae*^30^.

Presence of prophage genes obviously does not imply the functionality of the gene. Previously, experimental evidence of prophage-mediated virulence was demonstrated in environmental *V*. *harveyi* strains, which transformed into virulent forms following infection with a temperate phage^12^. However, prophage functionality determined by *in silico* predictions requires experimental validation, including induction experiments, gene expression studies and cytotoxicity evaluation, to assess the functional implications of prophage encoded properties and their dissemination in marine *Vibrio* communities.

The observed spacer match from the CRISPR region in *V*. *anguillarum* strain PF7 with a *zot*-encoding prophage in *V*. *anguillarum* strain PF4 (Fig. 6) indicated that CRISPR is used as an adaptive immunity defense against inoviruses in *V*. *anguillarum*, potentially affecting the evolution and virulence of this species^31^. The selection for defense mechanisms against *Inoviridae* infection emphasizes that the negative effects of phage inovirus infection may exceed the potential benefits from acquisition of virulence and other fitness factors. Thus, fitness cost such as replication of the additional phage DNA or interference with the fine-tuned physiology of the recipient cell^32^, may select against integration of inoviruses at certain conditions or in certain species.

While the present study provides a bioinformatic approach to assessing the potential importance of temperate phages for pathogenicity of *Vibrio*, some limitations to the analysis must be considered. First, the number of prophage-like elements likely represents a minimum estimate as the large number of phage-ORFs which are not related to known phage genes make prophages less recognizable by *in silico* analysis^33^. Second, some old *Vibrio* genomes were compromised by many assembly contigs (>200; See Supplementary Table S1); thus, prophage-like elements may have been split into multiple contigs and thus not detected. Finally, a high fraction of the prophage-like elements ranged from 5 to 10 kb (37.5% which were not related to *zot*-encoding prophages), and 56% of the sequences were incomplete prophages (Fig. 1A–D), indicating that the majority of *Vibrio* prophages probably have gone through mutational decay after integration and may lack the ability to lyse the cell and disseminate the phage-encoded genes to other *Vibrio* hosts. Despite these limitations, we consider this study as a starting point for further exploration of the ecology and evolution of *Vibrio* pathogens in aquatic systems. Lysogenic conversion in vibrios could represent a direct concern for human health, food safety and aquaculture industry^34,35^, and insight into the influence of phages as carriers of these virulence factors and as vehicles for their dispersal is essential for understanding the role of prophages as drivers of virulence in marine *Vibrio* communities.

## Methods

### Genome sequences collection

The *Vibrio* DNA sequences used in this study were obtained from National Center for Biotechnology Information database (NCBI) (October, 2016). A total of 1880 genomes representing sixty-four *Vibrio* species covered a variety of environments and wide geographic (>30,000 km) and temporal scales (>100 years) of isolation (Table S1, for genome details). We filtered out low quality assemblies which had N_50_ scores of <10 kbp and/or consisted of more than 400 contigs. Moreover, we included a collection of thirteen bacteriophage genome sequences belonging to the family *Inoviridae*. Accession numbers for each individual selected genome sequence were included in the Supplementary Table S1.

### Prophage-like element database construction

Prophage-like sequences were identified and selected by running available bacterial genomes in PHASTER (PHAge Search Tool)^36^. Because of their small sizes (typically between 5 to10 kb), some inoviruses were detected using a manual procedure by searching for similarity to known filamentous phages (See Supplementary Table S1 for phage genome details) by BLASTP (requiring an e-value <0.001). When at least three core ORFs from *inoviridae* family (Relative to CTX prophage: ADE80683.1 [*RstA*]; AAF29545.1 [*OrfU*]; YP_004286238.1 [*ace*]) were found in a window of 5–10 kb, reannotation of putative prophage ORFs was done by performing a BLASTP search against GenBank database^37^. The beginning and end of a specific prophage genome was estimated based on the annotation of the surrounding genes.

Output FASTA files containing prophage nucleotide sequences were subsequently subjected to a final manual review, including information about *Vibrio* species, specific strain and completeness (Complete, questionable or incomplete). Assessment of the completeness of the prophage was based on three specific criteria. Firstly, the genomic similarity of phage-related genes with prophage sequences deposited in the PHASTER database. Secondly, the presence of phage-related genes in a DNA sequence should be >50% of the total ORFs. Thirdly, the presence of specific phage-related cornerstone proteins (Integrase, fiber, tail, capsid, terminase, protease and lysin), attachment sites, tRNA or short nucleotides repeats should give a score of 10 for each key gene found. Based on these criteria a score value was calculated for each prophage sequence. A specific DNA sequence was considered a complete prophage-like element when the score was above 90 (See details^36^). Finally, the updated files were merged into a unique local custom database using Geneious V.10.1.3^38^. Although the current annotation was generally maintained, certain ORFs were re-analyzed using BLASTP and the annotation updated^37^. The *Vibrio* prophage-like elements database is available as MG-RAST database at library mgl583439.

Prophage-like element sequences were annotated by using MG-RAST server (version 3.3)^39^. The ORFs were distributed into different categories, according to similarity with protein databases.

### Identification of orthologs and ecologically relevant genes in prophage-like elements

The *Vibrio* prophage-like element database was screened for virulence factors, antibiotic resistance, niche adaptation and metabolic genes by tblastn alignment of annotated ORFs using BLAST + v2.2.24, with default parameters (E-value 10^−4^, amino acid identity >30%). Also, virulence or fitness factors previously identified in the fish pathogen *V*. *anguillarum*^7^ and *V*. *parahaemolyticus*^27^ were included in this study. Specific proteins in each of the orthologous groups were manually inspected using BLAST^40^ and Phyre2^41^.

### Phylogenetic analyses of Zot-like proteins

To reveal the phylogenetic relationship among genes encoding the identified zonula occludens toxins (Zot), we selected the 702 *zot* genes from complete inovirus sequences in our prophage-like element database. Deduced Zot amino acid sequences were aligned using Clustal W version 2.0^42^ and phylogeny was inferred using Maximum Likelihood (1,000 bootstrap replicates) in Geneious version 10.1.3^38^.

### CRISPR array identification

In *V*. *anguillarum*, CRISPR arrays had previously been identified and analyzed^22^. Repeat sequences were compared by WebLogo analysis, a Web-based application that generates graphical representations (logos) of the patterns within a multiple-sequence alignment^43^. Spacer sequences were aligned to the prophage-like elements using ClustalW^42^ in Geneious v.10.1.3^38^.

## Electronic supplementary material

Supplementary 1

Supplementary 2
